# Editorial: Increasing importance of patients-generated real world data for healthcare policy decisions about medicinal products: volume III

**DOI:** 10.3389/fphar.2026.1931444

**Published:** 2026-07-24

**Authors:** Wai Yee Choon, Kenneth K. C. Lee, Paul Scuffham, Shu Chuen Li, David Bin-Chia Wu, Jeff Jianfei Guo

**Affiliations:** 1 School of Pharmacy, Faculty of Medical and Life Sciences, Sunway University, Bandar Sunway, Malaysia; 2 School of Medicine and Health Sciences, Monash University Malaysia, Bandar Sunway, Malaysia; 3 School of Medicine and Dentistry, Griffith Health, Griffith University, Gold Coast, QLD, Australia; 4 School of Biomedical Sciences and Pharmacy, The University of Newcastle, Callaghan, NSW, Australia; 5 Saw Swee Hock School of Public Health, National University of Singapore, Singapore, Singapore; 6 Division of Pharmacy Practice and Administrative Sciences, James L. Winkle College of Pharmacy, University of Cincinnati, Cincinnati, OH, United States

**Keywords:** evidence-based access, health technology assessment, healthcare policy, patient preferences, patient-centric approach, patient-generated data, real-world data (RWD), real-world evidence (RWE)

## Introduction

Real-world data (RWD) and the real-world evidence (RWE) derived from it have moved from the margins to the centre of decision-making about medicinal products (Miksad and Abernethy, 2024). Over the past decade, both the European Medicines Agency (EMA) and the United States Food and Drug Administration (FDA) have developed dedicated frameworks for integrating RWE into regulatory submissions, reflecting a recognition that evidence generated outside controlled trial settings can address questions that trials alone cannot. It is of utmost importance that RWE is not replacing randomized controlled trials (RCTs) but rather becoming an increasingly important component of evidence-based decision-making although evidence on the safety, effectiveness and value of medicines is traditionally generated mainly from RCTs (Lambrelli et al., 2024; European Medicines Agency, 2023). There is growing consensus that appropriately conducted RWD studies can complement RCTs and fill critical evidence gaps, particularly in populations, settings, and time horizons that trials rarely accommodate, including long-term outcomes, real-world effectiveness, and comparative safety in patients with multimorbidity. Today, regulators, health technology assessment (HTA) bodies and payers increasingly draw on data generated in everyday clinical practice (Miksad and Abernethy, 2024; Lambrelli et al., 2024; European Medicines Agency, 2023) a trend reinforced by the EMA’s 2023 RWE framework and by a proliferation of guidance from regulatory and HTA agencies across North America, Europe, and the Asia-Pacific region, and, crucially, on data and preferences contributed by patients themselves, with patient-reported outcomes and patient experience data now increasingly embedded in submissions to regulatory and HTA bodies worldwide, to address questions that RCTs leave unanswered (Maruszczyk et al., 2022; Bertelsen et al., 2025). This third volume of the series continues a conversation that earlier volumes began: how can patient-centric methods turn the growing abundance of routine and patient-generated data into evidence that genuinely improves policy and care?

The Research Topic set out to assemble work that applies patient-centric methodologies across the medicinal-product life cycle: the design of studies using RWD and patient-generated data, the analysis of patient subgroups, outcome measurement that reflects patient experience and preference, integration of patient data with disease registries, and stakeholder collaboration that converts data into decisions. The seventeen contributing articles span methodological translation, the elicitation of patient preferences, comparative effectiveness and safety, evidence synthesis, economic modelling, and the evaluation of national policy. Collectively they illustrate both the promise of RWD and the practical, methodological and governance challenges that determine whether that promise is realised in real life practice.

## From real-world data to actionable evidence for assessment, regulation and access

A recurring message across the volume is that generating RWD is only the first step; the harder task is making it usable for those who make the decisions. Björvang et al. examine how academic RWD research produced within the European HTx project translates into practical HTA, drawing on case studies across disease areas, treatments and data sources to show where academic methods meet, and fall short of, the operational needs of assessment agencies. Sukkarieh et al. turn to the systemic barriers that constrain RWE uptake for drug regulatory affairs and market access in Saudi Arabia, mapping the data-quality, capacity and governance obstacles common to emerging RWE ecosystems. Policy itself becomes the object of study in the interrupted time-series analysis by Yu et al., which quantifies how China’s national volume-based procurement programme reshaped the use and expenditure of platinum-based antineoplastic drugs. Together these papers connect data infrastructure to the institutions that must act on it. A point to note is that policy evaluation should consider not only utilization and expenditure but also patient access, patient outcomes, quality of care, and system sustainability.

## Centring the patient voice

Patient-generated insight matters most when it captures what patients themselves value. Desmet et al. elicit the preferences of patients with Duchenne muscular dystrophy and their caregivers regarding gene therapy, to develo the attributes that should anchor future quantitative preference studies and, ultimately benefit–risk and reimbursement decisions for high-cost therapies. From a different vantage point, Assame et al. synthesise the factors that shape patients’ decisions to bring clinical-negligence claims against the NHS in England—a reminder that patient-generated information extends beyond clinical outcomes to experiences of care, trust and redress that carry their own policy weight.

## Mining routine and registry data for comparative effectiveness, safety and decision support

A large group of contributions mines routinely collected clinical and administrative data to compare therapies and surface signals that controlled trials may miss. Yu et al. apply propensity-score matching to intensive-care records and link pre-ICU statin therapy with lower 28-day mortality in sepsis-associated brain dysfunction, while Piao et al. use the MIMIC-IV database and inverse-probability-of-treatment weighting to associate early acetaminophen use with reduced 90-day mortality in ICU patients with ischaemic stroke. Staying with stroke, Xu et al. examine ondansetron as a candidate for improving clinical outcomes. Yeh et al. draw on a large US federated network to compare major adverse cardiovascular events in patients with co-occurring type 2 diabetes and schizophrenia treated with aripiprazole versus risperidone, illustrating how real-world cohorts can probe the comparative safety of competing agents in the multimorbid populations that trials often exclude. Two studies turn routine data into prospective decision support: Yang et al. develop and externally validate a machine-learning model to flag the risk of potentially inappropriate medication in elderly stroke patients, and Han et al. evaluate a multidisciplinary administrative–professional–technical programme to optimise antibiotic use and curb resistance in a tertiary hospital, an intervention that is itself a unit of health policy. Together these papers show both the reach of observational and data-driven methods and the confounding-related caution their interpretation demands.

## Synthesising evidence, modelling value and benchmarking with trials

Real-world decisions also rest on the rigorous synthesis of existing evidence and on modelling of long-term value. Jin et al. pool randomized data in a meta-analysis comparing the efficacy and safety of teriparatide with bisphosphonates in osteoporosis, and Yang et al. aggregate 51 trials to quantify the serum-uric-acid-lowering effect of SGLT2 inhibitors, identifying differences between agents that bear on drug selection in patients with cardiometabolic risk. Where randomized evidence is scarce, Liu et al. apply a single-arm rate meta-analysis to characterise the efficacy and safety of oral minoxidil in alopecia, pooling outcomes across many small studies. Economic value is addressed by Yan et al., who model the long-term recurrences avoided and the associated costs of adjuvant alectinib in Chinese patients with early-stage ALK-positive non-small-cell lung cancer, translating clinical benefit into the budgetary terms decision-makers require. Finally, two randomized controlled trials sharpen the comparators against which real-world studies are interpreted: Sun et al. report faster onset of sedation and higher recovery rates with remimazolam than midazolam during spinal-anaesthesia puncture, and Liu et al. find a shorter time to extubation with remimazolam besylate than midazolam in critically ill, mechanically ventilated patients. By defining effectiveness and safety under controlled conditions, such trials provide benchmarks for the routine-practice data examined elsewhere in this volume.

## Concluding remarks

Read together, the contributions to this volume trace the full arc from data generation to decision: building and translating the methods, eliciting the patient voice, comparing therapies in real-world populations, synthesising and modelling the evidence, and anchoring all of this to robust experimental benchmarks. But they also show that much work is still to be done. Data quality and completeness, unmeasured confounding in observational designs, the transferability of evidence across health systems, and the translation of patient preferences into formal assessment all remain real challenges. Tackling them adequately will require continued collaboration among patients, clinicians, methodologists, regulators and payers - the collaboration this series seeks to encourage. We thank the authors and reviewers for their work and efforts, and fittingly for a Research Topic built on patient-generated data, the patients whose experiences give this evidence its purpose and the decisions it informs their weight.
